# Sensitivity of genomic selection to using different prior distributions

**DOI:** 10.1186/1753-6561-4-S1-S5

**Published:** 2010-03-31

**Authors:** Klara L Verbyla, Philip J Bowman, Ben J Hayes, Michael E Goddard

**Affiliations:** 1 Animal Breeding and Genomics Centre, ASG Wageningen UR, PO Box 65, 8200 AB Lelystad, The Netherlands; 2 Biosciences Research Division, Department of Primary Industries Victoria, 1 Park Drive, Bundoora 3083, Australia; 3 Melbourne School of Land and Environment, The University of Melbourne, Parkville 3010, Australia; 4 The Cooperative Research Centre for Beef Genetic Technologies, University of New England, Armidale, NSW 2351, Australia

## Abstract

Genomic selection describes a selection strategy based on genomic estimated breeding values (GEBV) predicted from dense genetic markers such as single nucleotide polymorphism (SNP) data. Different Bayesian models have been suggested to derive the prediction equation, with the main difference centred around the specification of the prior distributions.

**Methods:**

The simulated dataset of the 13^th^ QTL-MAS workshop was analysed using four Bayesian approaches to predict GEBV for animals without phenotypic information. Different prior distributions were assumed to assess their affect on the accuracy of the predicted GEBV.

**Conclusion:**

All methods produced GEBV that were highly correlated with the true breeding values. The models appear relatively insensitive to the choice of prior distributions for QTL-MAS data set and this is consistent with uniformity of performance of different methods found in real data.

## Background

Genomic selection describes a technique for evaluating an animal's breeding value by simultaneously evaluating and summing marker effects across the genome. It uses panels of SNPs covering the whole genome so that ideally all QTL are in linkage disequilibrium with at least one marker, thereby maximizing the proportion of genetic variance explained by the SNPs.

Meuwissen et al (2001) [1] presented three models to produce GEBV. The first invoked the infinitesimal model assumption such that all SNPs had effects derived from the same normal distribution. The other approaches used a Bayesian framework to apply hierarchical models with different prior distributions assuming unequal variances across the SNP, resulting in a *t* distribution for prior distribution for the QTL effects. The specification of the prior distributions of the QTL effects has been reported to be important to the accurate prediction of breeding values and when mapping multiple QTL across the entire genome [2].

The aim of this study was to assess the effect that different prior distributions and subsequently the models using these priors, had on the accuracy of estimated GEBV using the 13^th^ QTL-MAS simulated data set where we had no prior knowledge of the trait's distribution of QTL effects.

## Methods

### Model

At each loci (total number of locus, p) there are three possible combinations of two alleles (e.g. A or B), the homozygote of one allele (AA), the heterozygote (AB) and the homozygote of the other allele (BB). These are then quantitatively represented by 0, 1 and 2 respectively. Subsequently, phenotypic records at each time point were modelled as:

where *y* is the vector of phenotypes of the trait being analysed for all n individuals, μ is the mean, ***1_n_*** is a vector of ones of length n, **X*_j_*** is a vector of indicator variables representing the genotypes of the *j^th^* marker for all individuals (*x_ij_*=0,1,2), *β_j_* is the size of the QTL effect associated with marker *j*, **u** is the vector of random polygenic effects of length *n* (*Z* is the associated design matrix) and is assumed to be normally distributed, ***u*** ~ ***N*** (0, ***A***) where*** A*** is the pedigree derived additive genetic relationship matrix and **e** is the residual error also assumed to be normally distributed, ***e*** ~ ***N***(0, ***I***) where ***I*** is the *n*x*n* identity matrix. The prior distributions for the variances of the random polygenic effects and the residual were uninformative flat priors of the form *Χ*^-2^(- 2,0). The GEBV at each time point were calculated as

### Prior distributions for SNP effects and algorithms

Four differing sets of prior distributions were assessed and the specifications are shown in Table 1. The Bayes BLUP model assumed the same variance for the normal distribution from which the SNP effects were assumed to be derived (maintaining the infinitesimal assumptions for traditional BLUP). The variance of the normal distribution was sampled once every MCMC iteration using a Gibbs Sampler. The SNP effects were subsequently sampled from this normal distribution. The model termed Bayes A [1] assumes that the SNP effects come from a *t*-distribution. This is because an efficient Gibbs sampling scheme to sample the SNP effects from their posterior distributions is to a sample SNP specific variance from an inverse chi-square distribution, then use this variance to define the normal distribution from which the SNP effect is sampled [1]. The values for the inverse scaled chi square hyper parameters( *r* and *S*) were calculated as in Meuwissen et al (2001) [1].

**Table 1:** Prior Distribution Specifications

Method	Prior Distribution
Bayes BLUP	

Bayes A	

Bayes A/B (Hybrid)	with probability 1- π with probability π

Bayes C	*γ _i_* ~*bernoulli*(π) 1 - p(*γ _i_* = 0) = *p*(*γ _i_* = 1) = π

*β_i_* is the effect for the i^th^ SNP and *γ _i_* is the indicator variable for the i^th^ SNP.

The other two models assumed mixture distributions for the SNP effects reflecting the assumption that there is a large number of SNPs with zero or near zero effects and a second smaller set of SNPs with larger significant effects. A Bayes A/B "hybrid" method was used. This approximation to Bayes B [1] was used to keep computational and time demands reasonable. In this algorithm, after every k Bayes A iterations, Bayes B via the reverse jump algorithm is employed. The Reverse Jump algorithm [3] is run multiple times per SNP and then any SNP with a final state of zero in the current Bayes B iterations is set to zero for the subsequent k iterations of the Bayes A. This maintains the correct transitions between models of differing dimensionality. The prior distributions are identical to that of the original Bayes B using a mixture prior distribution for the SNP variance allowing a proportion, 1-π, to be set to zero. The other proportion π is sampled from the same mixture distribution as Bayes A. See Meuwissen et al (2001) for more details of priors and conditional distributions used.

A faster alternative to both the Bayes A/B hybrid and Bayes B is to use Stochastic Search Variable Selection (SSVS) [4] (Bayes C [5,6]). This avoids the problem of the changing dimensionally of the models by providing a technique to maintain constant dimensionality across all models while still allowing the SNP in the predictive set to change. Instead of removing all non-significant parameters, their posterior distributions are limited to values close to zero. The major advantage of this method is that it can be implemented using the Gibbs sampler instead of the more computationally demanding algorithms such as the reverse jump algorithm. The indicator variable (*γ_i_*) determines whether the i^th^ SNP effect is sampled from the larger distribution (i.e. significant effect) or from the small distribution with near zero effects (see Table 1). The prior values of π (the proportion sampled from the non-zero distribution or the larger distribution respectively) for both Bayes A\B and Bayes C was set to 0.05, reflecting the fact that with 435 SNP, it appeared reasonable to expect at least 21 SNP would be associated with a QTL.

The algorithms associated with each model were run for 30,000 iterations with the first 10,000 discarded as burn-in.

## Results and Discussion

### Prediction of breeding values at time point 600

The problem of how to model the time series data and estimate GEBV at time point 600 was explored. However, there was little information available to estimate any inflection points or asymptotic values. The GEBV estimated at time points 265, 397 and 530 were found to have a linear relationship (eg. appeared to form the linear part of the growth curve). Consequently, as there was no other information available after time point 530 to predict asymptotes etc., the GEBV at time point 600 were estimated by fitting a linear regression through the breeding values at the three linear time points (265, 397 and 530).

### Breeding values

The correlations between the GEBV (t=600) predicted by the alternative methods for the validation population containing the 50 full sib families without phenotypes are shown in Table 2. Correlations were extremely high between all methods other than BLUP and consequently GEBV appeared relatively insensitive to the model used when assuming unequal variances. Correlations, mean square errors, the accuracy of predicting the first 100 animals (rank) and the bias (regression coefficient) between the predicted and true breeding values are shown in Table 3. While there is no significant difference between the methods, Bayes A/B performed the best of the methods producing the lowest MSE, highest correlation and rank but was slightly more biased than Bayes C and Bayes BLUP, but not significantly. Interestingly while Bayes C has very similar hierarchical prior distributions it does worse than Bayes A/B. Further optimisation of the prior probability of π for Bayes C increased the accuracy (results not shown). The optimal value for π was 0.3 (values tested were 0.05, 0.1, 0.2, 0.3, 0.4, 0.6 and 1). This produced results more similar to the results seen for Bayes A\B. This does highlight the importance of the correct assumption of the proportion assigned to the smaller and larger distributions in a mixture model. This difference between these two methods may demonstrate that Bayes C is more sensitive to an incorrect assumption about this proportion.

**Table 2:** Correlations Between Estimated GEBV for unphenotyped animals at t=600

	Bayes C	Bayes A/B	Bayes BLUP
*Bayes A*	0.999	0.991	0.860
*Bayes C *	1	0.993	0.863
*Bayes A/B*		1	0.893

**Table 3:** Comparison of True and Estimated GEBV.

*Method*	*Correlation*	*MSE*	*Rank*	*Regression*
Bayes.BLUP	0.885	5.479	0.691	0.979
BayesA	0.857	7.092	0.696	1.162
BayesA/B	0.889	5.435	0.73	1.081
BayesC	0.861	6.561	0.71	1.024

Correlation coefficient between the true and predicted GEBV, Mean Square Error (MSE), Rank (Accuracy of the predicting the best 100 animals) and the Regression Coefficient of the true breeding value on the estimated GEBV.

The inclusion of the polygenic effect in the model (not simulated in the data) only slightly reduced the accuracy of prediction (.01) but not significantly (results not shown). It was included in the model as its inclusion has been shown to produce slightly better accuracies of prediction while reducing the bias of the variance components[7].

Bayes BLUP produced a significantly different set of GEBV. This is evident by the much lower correlations with the other methods and difference in regression coefficients between BLUP and the other methods. Despite these differences Bayes BLUP produces good accuracy and a low MSE (Table 3). Hayes et al (2009) [8] reports that New Zealand, Australian, the Netherlands and United States studies all found that BLUP gave lower accuracy of GEBV than Bayesian Methods for traits where there is a single QTL that explains a large proportion of the genetic variance e.g. DGAT1 for Fat Percentage. In the current dataset a finite number of QTL were simulated where the largest amount of genetic variance explained by a single QTL was 10.5%. Despite this, Bayes BLUP is still able to produce very accurate GEBV compared to the other methods. One reason this occurs may be that a number of SNPs are required to pick up the effect of a single QTL, resulting in large numbers of SNPs with small effects, which matches the prior distribution of BLUP. However if the percentage of genetic variance explained by a single QTL was to be larger, Bayes BLUP could be expected to produce worse results. Thus this caveat to using Bayes BLUP should be considered when using this method.

## Conclusion

All methods produced GEBV that were highly correlated (greater than 0.85) with the true breeding values despite diverse assumptions and prior distributions. This indicates that the hierarchical model is relatively insensitive to the choice of prior distributions for this data set. Thus all models perform well and this is consistent with the general uniformity of performance found across methods in real data. [8]. Despite the general equality in the performance of the different methods, it is still recommended that any information about a trait's QTL effect distribution and phenotypic data should be used to determine the choice of model, prior distributions and setting of the hyper parameters. This will maximise the likelihood of calculating the most accurate GEBV possible.

## Competing interests

The authors declare that they have no competing interests.

## Authors' contributions

KV carried out the analyses and drafted the manuscript. PB developed the Bayes A and Bayes BLUP software. KV created the Bayes C and Hybrid software using the Bayes A software. BH and MG read and suggested improvements to the manuscript. All authors read and approved the final manuscript.
